# Process evaluation of a complex intervention to promote advance care planning in community-dwelling older persons (the STADPLAN study)—study protocol

**DOI:** 10.1186/s13063-020-04529-2

**Published:** 2020-07-16

**Authors:** Katharina Silies, Rieke Schnakenberg, Almuth Berg, Änne Kirchner, Henriette Langner, Juliane Köberlein-Neu, Gabriele Meyer, Falk Hoffmann, Sascha Köpke

**Affiliations:** 1 Institute for Social Medicine and Epidemiology, Nursing Research Unit, University of Lübeck, Ratzeburger Allee 160, 23562 Lübeck, Germany; 2 Department for Health Services Research, Faculty of Medicine and Health Sciences, Carl von Ossietzky University Oldenburg, Ammerländer Heerstraße 140, 26129 Oldenburg, Germany; 3 Medical Faculty, Institute for Health- and Nursing Science, Martin Luther University Halle-Wittenberg, Magdeburger Straße 8, 06112 Halle (Saale), Germany; 4 Center for Health Economics and Health Services Research, Schumpeter School of Business and Economics, University of Wuppertal, Rainer-Gruenter-Straße 21, 42119 Wuppertal, Germany; 5 Institute for Nursing Science, University of Cologne, Gleueler Straße 176 – 178, 50935 Köln, Germany

**Keywords:** Process evaluation, Study protocol, Logic model, Complex intervention, Mixed methods, Home care setting, Ambulatory setting, Nursing, Advance care planning

## Abstract

**Background:**

Process evaluation addresses the implementation, mechanisms of impact, and context of participants in complex interventions. The STADPLAN study assesses the effects of conversations on advance care planning (ACP) led by trained nurse facilitators. The complex intervention consists of several components that may lead to various changes in attitude and behavior regarding personal ACP activities. With the process evaluation, we aim to assess how changes were achieved in the STADPLAN intervention.

**Methods:**

The planned process evaluation study will be conducted alongside a cluster-randomized controlled trial on ACP in home care services (HCS). Trained nurse facilitators will deliver the ACP intervention consisting of an information brochure and two ACP conversations. A logic model depicts the assumed change processes of the intervention: the educational program enables nurses to conduct ACP conversations with patients and their caregivers. Patients gain knowledge and reflect upon and engage in their own ACP. Caregivers better understand patients’ wishes and feel reassured in their role as surrogates. Designation of a surrogate and communication on ACP are facilitated. We will assess the effects of the educational program with questionnaires and a focus group including all participating nurses. We will measure ACP engagement, and prevalence of advance directives in patients, and ask for their experiences with the intervention. We will conduct semi-structured interviews with caregivers about their expectations and experiences regarding ACP in general and the intervention. We will address context factors, e.g., basic characteristics of the HCS (such as ownership, number of clients, staff and qualification). Analysis will be based upon the logic model, integrating qualitative and quantitative data.

**Discussion:**

The comprehensive process evaluation will provide essential information on the feasibility of implementation strategies and the clinical relevance of a nurse-led ACP intervention in home care recipients and its generalizability and transferability to other settings.

**Trial registration:**

German Clinical Trials Register: DRKS00016886. Registered retrospectively on June 4, 2019, first participant included on May 29, 2019.

## Contribution to the literature

- The STADPLAN study is a large cluster-randomized controlled trial and one of the first trials on ACP in community dwellers. It will provide evidence on the effectiveness of an ACP intervention provided by nurses in home care
- The process evaluation as outlined in this paper will indicate how the ACP intervention is related to patient activation, ACP engagement, and surrogate designation and evaluate its feasibility in the home care setting
- The mixed methods approach based on the MRC framework for the development and evaluation of complex interventions will ensure high methodological rigor
- Results will support the implementation of ACP in the community setting

## Background

### Advance care planning

“Advance care planning is a process that supports adults at any age or stage of health in understanding and sharing their personal values, life goals, and preferences regarding future medical care. The goal of advance care planning is to help ensure that people receive medical care that is consistent with their values, goals, and preferences during serious and chronic illness” [1].

ACP can be documented in written form and relatives can be involved in the communication [2–4]. The need for ACP arose in relation to evolving medical treatment options and the increased possibilities to prolong life. To retain patients’ autonomy despite unconsciousness or impaired decisional capacity has gained increasing weight. Legislation has been developed to protect patients’ rights and to avoid medical paternalism [5–7]. As these cannot sufficiently address patients’ needs, the concept of ACP evolved. Here, programs were developed that promoted reflection on personal values and communication with health professionals and within families [8, 9]. In Germany, advance directives are legally binding by law since 2009 (German Advance Directives Act [Patientenverfügungsgesetz]) and ACP conversations costs can be covered by the statutory health insurance for people living in nursing homes or facilities providing integration assistance for disabled people since 2015 (Hospice and Palliative Care Act [Hospiz- und Palliativgesetz]). Despite such legislation, ACP and advance directives (AD) have not been sufficiently implemented [10]. Therefore, we will conduct a cluster-randomized controlled trial assessing a complex intervention aiming to promote ACP in community-dwelling older persons (the STADPLAN study: STudy on ADvance care PLANning in care-dependent community dwelling older persons) [11].

### The STADPLAN study

In this study, we will adapt an ACP program to the German home care setting, which has been successfully implemented in other countries and settings [12, 13], among these also German nursing homes [9]. We will include 16 home care services (HCS) both in the intervention and the control group, with a total of 960 participating HCS clients. Trained nurse facilitators of the HCS will deliver ACP. The primary endpoint is patient activation assessed by the German version of the Patient Activation Measure (PAM-13) [14, 15]. The PAM-13 is a valid and reliable instrument assessing the degree to which individuals take an active role in managing their own health, the corresponding health care and its consequences, and the extent to which individuals feel competent to fulfill that role.

We will measure secondary endpoints, such as proportion of persons with advance directives, hospitalization, and quality of life as well as depression and anxiety.

The study protocol of the cluster-randomized trial has been published [11]. This paper outlines the comprehensive process evaluation conducted alongside the trial.

### Process evaluation in complex interventions

The complexity of interventions is determined by several dimensions, e.g., the number and interaction of components, the number and difficulty of behaviors required for delivering or receiving the intervention, or the number of groups or organizational levels targeted [16, 17]. This implies that the measurement of a single outcome on the level of one target group does not sufficiently depict how the intervention causes change and which factors influence the outcome. Yet, this knowledge is necessary to adapt interventions to other settings and groups. This means, that in complex interventions, outcomes have to be assessed at all steps of the intervention process and in all groups or participants involved, in order to find out whether and how an intervention will work in practice [17]. If complex interventions do not show the anticipated effects, the process evaluation aims to find the reason for this and how the intervention should be adapted to improve the intended outcomes.

According to the UK Medical Research Council’s framework for the development and evaluation of complex interventions, the first question to be answered is as follows: what is the theory behind the intervention? Why is the intervention supposed to work, in which participants and by which mechanisms [18]?

On this theoretical basis, intervention components, participants that are involved and context factors, as well as expected effects, can be depicted in a logic model [18, 19].

A logic model allows to allocate outcomes to participants and processes and thus forms the foundation for the development of the research design and the instruments of the process evaluation. Furthermore, contextual factors inherent to participants and organizations are considered, as they influence the procedural and overall outcomes.

The process evaluation aims to depict how the STADPLAN ACP intervention is embedded in the context of participating HCS, patients, and caregivers, how it was received and accepted, and whether it is feasible in the home care setting.

## Methods

### STADPLAN logic model

We developed and piloted the STADPLAN logic model. Taking necessary adaptations of the intervention to the German home care setting into account, we created and discussed the logic model within the collaborating group (all authors) to ensure that relevant actors and procedural outcomes were incorporated. In the pilot study, we tested the evaluation instruments for feasibility.

The focus of the process evaluation lies on all participants involved and their context, namely the HCS, nurses, patients (or clients) of the HCS and their caregivers (i.e., family caregivers or surrogates of the participating patients).

**Fig. 1:**
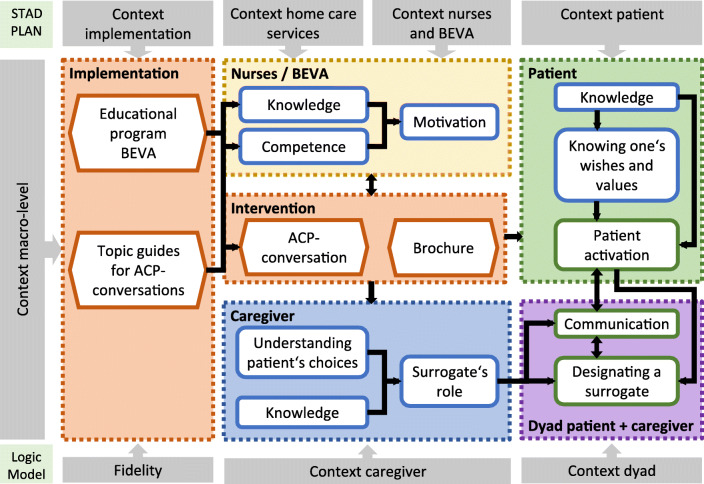
Components of the complex intervention—the STADPLAN logic model. BEVA, trained nurse facilitator; ACP, advance care planning; patient, participating client of the home care service; caregiver, family caregivers or surrogates of the participating patient

The logic model for the STADPLAN trial describes (1) the intervention, (2) the implementation, (3) the participants and their mutual relationship, (4) their context, and (5) the anticipated procedural outcomes on the individual level (see Fig. 1).

#### Intervention

The STADPLAN core intervention consists of two guided ACP conversations with patients and caregivers led by trained nurses (BEVA, German acronym for “facilitator for ACP in the home care setting”), including an information brochure with workbook.

During the first conversation, the BEVAs collect information on patients’ ACP activities to date and hand out the information brochure. BEVAs assess patients’ knowledge about ACP and provide additional information accordingly. They motivate patients to read the brochure and use the questions and commentary fields to reflect upon their thoughts regarding ACP. They invite patients to include one or more persons of trust, such as family members or designated surrogates (referred to as “caregivers”), to take part in the second conversation.

In this second conversation, BEVAs talk with the patients about their views on life, quality of life, life expectancy, situations of decisional incapability, and wishes regarding life-prolonging treatment.

The focus of the conversation is to support patients in reflecting about their wishes and values, to guide the involved persons of trust in listening to patients’ views and in their role as proxy decision maker, and to facilitate further conversation within the family. The aim of the intervention is thus to enable patients to act in their own ACP. The conversations follow structured topic guides, containing commentary fields which the BEVAs use during the conversations to document the discussed topics.

The information brochure with workbook contains information on ACP in plain language and contact details of regional advisory services. The workbook section contains questions facilitating reflection on ACP and room to write them down.

#### Implementation

Trained nurses of the participating HCS will conduct the intervention. Their training (educational program) encompasses two 1-day workshops, course material, the information brochure, and topic guides with commentary fields for ACP conversations.

The first workshop provides knowledge on ACP, types of AD (advance directives), surrogate decision-making, and training on the use of the topic guides, which support BEVAs in leading the conversation and ensure that all relevant issues are discussed. Furthermore, the topic guides serve as documentation for adherence, as BEVAs are instructed to use all commentary fields to document what was discussed.

The second workshop is designed to refresh content of the first workshop and to address BEVAs’ experiences with ACP conversations. It is therefore tailored to the BEVAs’ needs, based on structured modules. Before we finally determine which modules to address during the workshop, we will question BEVAs via phone about their experiences with the ACP conversations and suggestions to identify workshop topics. Based on experiences of the pilot study, these could be repetition of informational content; simulation of difficult conversations; peer discussion of experiences regarding recruitment, organization, or challenging interview situations; or other topics requested by the BEVAs. At the end of the workshop day, we will reflect on BEVAs experiences and discuss their overall assessment of the study in a focus group.

#### Participants

Participants of the complex intervention are patients, BEVAs, and caregivers. Patients are clients of the participating HCS, 60 years or older, and care dependent (based on assessment by the long-term care insurance). Caregivers are family members who are defined by patients as their main carers. BEVAs are registered nurses of the participating HCS.

#### Context

To describe contextual factors is essential in complex interventions, as these can affect the implementation and mechanisms of impact of the intervention, thus potentially influencing the targeted outcomes. Contextual factors differ, depending on the setting of an intervention, and have to be addressed specifically tailored to the intervention and setting to be evaluated [18]. Different context perspectives related to all participants and levels of the core intervention can be determined in the STADPLAN study.

- Context of the HCS: basic characteristics, resources, organizational norms regarding ACP
- Context of nurses and BEVAs: experiences, attitudes, expectations that are relevant for the intervention
- Context of patients and caregivers: experiences, attitudes, expectations, relationship within the dyad and with the HCS
- Context of the implementation and the intervention: organization and conduct of the educational program and ACP conversations
- Context macro-level: actual and perceived norms regarding ACP in the home care setting in general, new developments regarding ACP (like legislation or changes in funding), other incidents affecting the daily practice of HCS with potential influence on the study procedures

#### Anticipated procedural outcomes

We will assess procedural outcomes primarily on the individual level, addressing, e.g., knowledge, attitudes, and self-perceived competencies. The educational program enables and motivates nurses to conduct meaningful ACP conversations with patients and their caregivers. Patients learn about ACP and reflect upon what matters to them in life and what this implies for end-of-life care. They feel prepared to actively engage in their own ACP. Caregivers gain knowledge on ACP and a better understanding of patients’ wishes and attitudes. Thus, they feel reassured in their role as surrogates. The intervention facilitates the designation of a surrogate and the communication on ACP within the patient/caregiver dyad.

### Process evaluation framework and methods

**Fig. 2:**
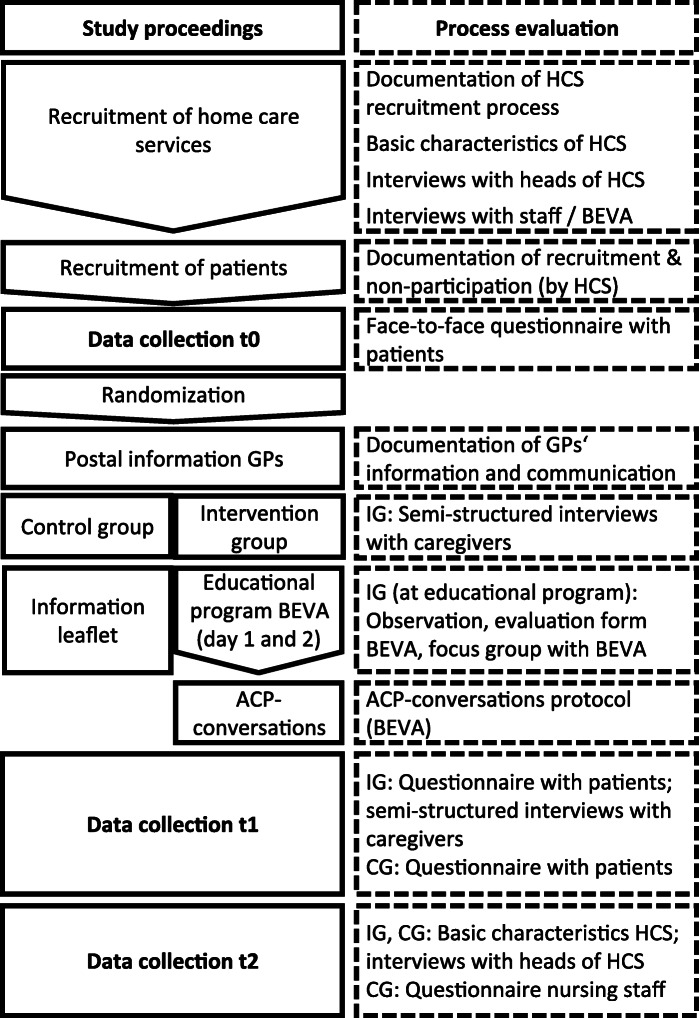
Flowchart of the STADPLAN study and process evaluation. HCS, home care service; GP, general practitioner; BEVA, trained nurse; IG, intervention group; CG, control group; ACP, advance care planning; nursing staff, nurses in the control group who will take part in a 1-day workshop offered to control group HCS

The process evaluation follows the course of the main study as depicted in Fig. 2. We chose a mixed methods approach to allow for findings, we did not anticipate emerging, and we will be able to link qualitative with quantitative outcomes, thus enriching our results. Data will be collected at baseline (t0), at the second day of the educational program, and at t1 (6 months after baseline) and t2 (12 months after baseline) (see Table 1). Baseline is defined on the individual patient’s level.

**Table 1:**
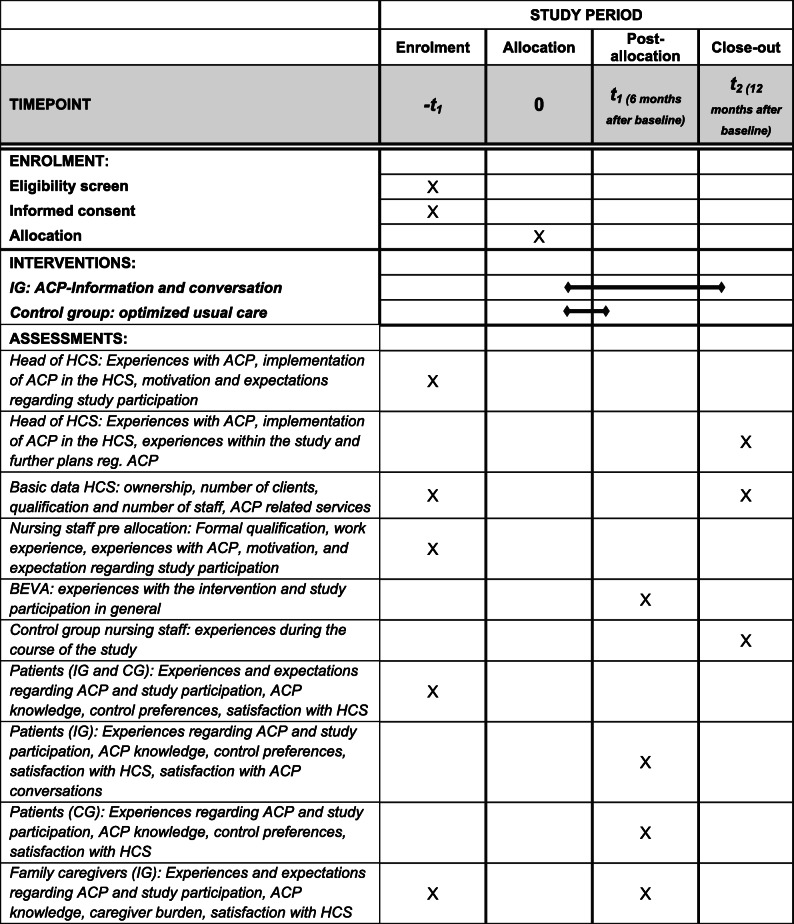
Process evaluation schedule of enrolment, interventions, and assessments

*ACP* advance care planning, *optimized usual care* control group participants receive short written information on ACP, *HCS* health care service, *BEVA* trained nurse facilitator for ACP conversations, *IG* intervention group, *CG* control group

>

**Fig. 3:**
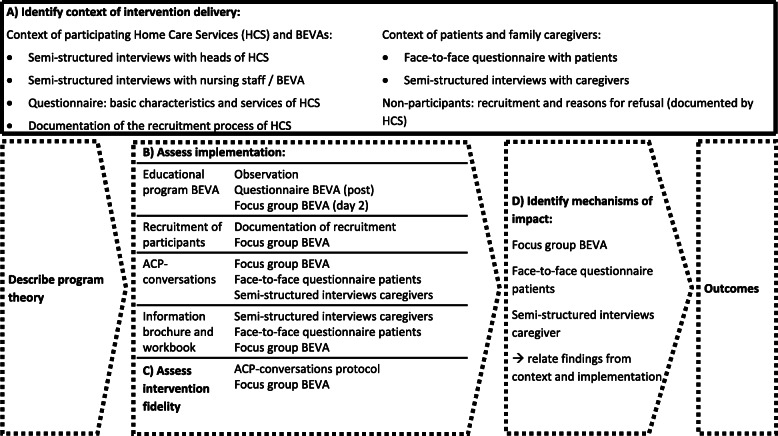
Process evaluation framework and methods, adapted from Moore et al. HCS, home care service; BEVA, trained nurse facilitator

We will use a variety of instruments to capture intervention processes. The process evaluation is based on the MRC framework on process evaluations for complex interventions [18] as shown in Fig. 3.

#### Context of the intervention delivery

To describe the context of the participating HCS, we will use qualitative interviews with the heads of the HCS and nursing staff and assess basic characteristics such as ownership, number of clients, and number and qualification of staff quantitatively [20].

To gauge overall prerequisites for the conduct of our study in the home care setting, we will transparently describe the recruitment process. Time constraints, staff shortage, and scarce funding might hinder HCS to participate in the study and influence the study’s progress. We will therefore document non-participation of all invited HCS during the recruitment process in a standardized template at each study site. The HCS will anonymously document the recruitment of participants with standardized forms and question non-participants on patients’ level for their reasons to decline. We will categorize reasons for non-participation on HCS and patient level and report them descriptively. Furthermore, we will discuss recruitment obstacles within a focus group on day 2 of the educational program with the present BEVAs.

We will conduct face-to-face interviews with closed and open-ended questions with patients, concerning their knowledge and expectations regarding ACP [21, 22], their engagement in ACP [23], control preferences [24], and their satisfaction with the HCS [25]. The pilot study showed that these data take additional 10–15 min to collect, which is only feasible with patients who manage the main data collection well. We therefore decided to achieve a convenience sample of 128 patients (4 per cluster).

We will conduct semi-structured interviews with caregivers about their experiences and expectations regarding ACP [26], their satisfaction with the HCS [25], and how they experience their situation as caregiver [27–29]. We aim to recruit a convenience sample of one participant per intervention cluster (*n* = 16) asking patients and caregivers present in the main data collection for participation in a separate telephone interview.

#### Implementation

We will focus on four elements of the intervention: the educational program; the recruitment of participants; the ACP conversations; and the information brochure with workbook. We will document the conduct of the educational program by observations focusing on adherence and involvement/interaction of participants. We will base the observations on a template listing each activity of the educational program. The observers (research assistants) will document length and method of the activity, involvement of participants, and questions/unexpected events. The outcome on BEVAs’ level will be assessed with questionnaires at the end of the respective program day, testing knowledge on ACP and self-reported confidence in communication abilities, and expected feasibility of the upcoming ACP conversations in all participating BEVAs. At the second workshop day of the program, we will conduct a focus group discussion on the experiences of the nurse facilitators, as this will take place after completion of most of the ACP conversations. We aim to include all BEVAs present at the workshop, yet participation is voluntary. We will audiotape the focus group discussion and transcribe verbatim using transcription rules adjusted to the aim of content analysis.

HCS will document recruitment of participants as described above.

We will evaluate the ACP conversations on the level of BEVAs (in the focus group discussion), on the level of patients at t1 using face-to-face questionnaires with open-ended questions, and on the level of caregivers using qualitative telephone interviews. This multi-dimensional approach allows for gaining a comprehensive picture of all involved parties’ experiences. The same approach will serve to evaluate the information brochure with workbook.

#### Implementation fidelity

We will use the documentation of the BEVAs’ ACP conversations to estimate adherence to the planned procedure. The topic guide forms provide commentary fields and BEVAs are instructed to document whether the questions were discussed and concerning which content or result. BEVAs’ experiences with the topic guides, feasibility, and reasons for non-adherence will also be discussed in the focus groups.

#### Mechanisms of impact

Mechanisms of impact describe reciprocal influence and interaction of participants and intervention. In this case, this means the influence of the ACP conversations on patients, caregivers, and BEVAs and how this shapes the conduction of the intervention. We will evaluate these mechanisms at t1 in patients with open-ended questions regarding their experiences and fulfillment of expectations as well as their perception of BEVAs’ performance (face-to-face questionnaire patients, *n* = 64, intervention group). We will interview caregivers by phone at t1, focusing on individual experiences, perception of BEVAs’ performance, and perceived benefits or pitfalls of the intervention (*n* = 16, intervention group). We will assess impact on the communication about ACP in the family and ACP engagement in all patients as part of the main data collection at t0–t2. In the semi-structured interviews, we will ask caregivers about habits of decision-making within the dyad at t0 and t1.

### Recruitment and data collection

We aim to recruit patients’ caregivers or family members, heads of HCS, and BEVAs/nursing staff of the control group, related to all HCS (see Table 2, recruitment goals). We will obtain written consent and interview patients in conjunction with the main data collection visits at their homes. We will interview heads of HCS and nursing staff/BEVA face-to-face or by phone. At t1, we will perform group-specific questionnaires with patients and interviews with caregivers separately via phone. Informed consent will be sought from all participants and documented. Experiences from the pilot study indicated that recruitment would take several months. Therefore, baseline has been defined on individual participants’ level. T1 and t2 take place 6 and 12 months after baseline.

**Table 2:** Recruitment goals

Participant	***N*** at t0	***N*** at t1	***N*** at t2	Method
Head of HCS	32		32	Semi-structured interview
BEVA/control group nursing staff	32			Semi-structured interview
BEVA (IG)		3		Focus group (t1/day 2 workshop, one per study site)
Nursing staff (CG)			16	Questionnaire (telephone)
Patients (IG + CG)	128			Face-to-face questionnaire
Patients (IG)		64		Face-to-face questionnaire IG
Patients (CG)		64		Face-to-face questionnaire CG
Family caregivers (IG)	16	16		Semi-structured interview

*HCS* home care service, *BEVA* trained nurse, *IG* intervention group, *CG* control group

We will conduct semi-structured interviews face-to-face or by phone and audiotape and transcribe interviews verbatim, anonymizing data in the transcription process. We will store all data for the process evaluation in an anonymized manner, and document only study site and date/measurement point on data sheets, as we decided against linking the process data and the main outcome on an individual level and opted for a cumulated comparison between the points of assessment at baseline/enrolment and t1, and between control group and intervention group. We will use data necessary to contact patients and family caregivers at home only to access them for scheduling and conducting data collection. During our pilot study, we tested the interviews with caregivers (*n* = 5), patients (*n* = 5), heads of HCS (*n* = 4), and staff (*n* = 3). Only minor revisions were necessary where we deemed questions redundant.

### Data analysis

We will analyze data from all semi-structured interviews using the program MAXQDA Standard 12 (Release 12.3.5, VERBI GmbH Berlin). We will conduct a content analysis and use a deductive–inductive approach [30]. Based on the constructs of the logic model, we will code the data, allowing new findings to emerge and to be integrated. By summarizing the perspectives of the different participants on the level of the logic model as coding scheme, we will be able to depict all constructs from several points of view. We will analyze quantitative data descriptively using IBM SPSS Statistics (Version 22.0.0.1) and integrate findings into the structure of the qualitative analysis. We developed an analysis plan, associating the qualitative and quantitative variables with the constructs of the logic model. We will provide a codebook in which the variables of the mixed methods are linked to the codes.

### Ethics

ACP requires reflecting on personal values, end-of-life care, and death. These topics must be treated in a sensitive and respectful manner, as they may cause distress in participants. Data collection, especially semi-structured interviews, will be conducted by experienced research assistants, who are not involved in patients’ care and have no professional or personal relationship with the patient. Interviews with patients will be conducted at their own homes in familiar environment. Patients will receive written information about the study and will have the possibility to ask questions before giving written consent. They will be informed about the possibility to end participation at any time without negative consequences regarding their care or relationship towards the HCS. Heads and BEVAs of the collaborating HCS and caregivers will receive oral information on the aim of the data collection, anonymization, data storage, dissemination, and voluntary consent. The have the possibility to ask questions and give oral consent or decline without negative consequences.

## Trial status

Protocol version: Version 3 – 29.12.2019.

The STADPLAN Trial is currently being implemented and will be completed in January 2021. Recruitment started on May 29, 2019, and will be completed by January 31, 2020.

## Discussion

The STADPLAN study is a large cluster-randomized controlled trial and one of the first trials in ACP in community dwellers. It will provide evidence on the effectiveness of an ACP intervention provided by trained nurses in home care. The comprehensive process evaluation will indicate how the ACP intervention is related to patient activation, ACP engagement, and surrogate designation and evaluate its feasibility in the home care setting. We will follow current internationally acknowledged guidelines [18]; the process evaluation is founded on a theory-based logic model. Yet, it will not be possible to illuminate every process in detail. For example, we will use the BEVAs’ documentation to estimate how closely the planned procedure in the ACP conversations was followed and ask participants for their experiences. These surrogate parameters will indicate to which degree the intervention was implemented as intended. We refrained from observing or audiotaping the conversations, as ACP is a sensitive topic, the conversations should be confidential, and participants (including patients, caregivers, and BEVAs) need to feel comfortable. Patients show varying levels of cognitive capacity and exhaustibility, so we decided to use a convenience sample, taking patients’ individual conditions into account and asking for consent to conduct extra questionnaires. This might introduce a selection bias, e.g., for ACP knowledge, or satisfaction with the HCS, but we chose to accept this rather than losing participants during data collection. Therefore, we pre-tested instruments and processes which have proven to be feasible in elderly participants.

Process evaluation is of utmost importance, but still it must be feasible. The MRC framework recommends to rather reduce the amount of data collected in favor of collecting the right data. For this reason, we developed the logic model within the project group, which provides guidance and commitment on the relevant questions.

Looking at the key recommendations for process evaluation by the MRC framework [18], the strengths of the STADPLAN process evaluation can be described accordingly:

### Planning of the process evaluation

For this phase, the key recommendations focus on the relationship, the expertise, and the synchronization of the process and outcome evaluation teams.

Researchers of four universities with experiences in successful earlier study collaborations conduct the STADPLAN study. We developed the intervention and the process evaluation in close cooperation but with different responsibility among partners. All partners discussed the logic model intensively, to make sure that it represents the linking key.

Thus, the close relationship, yet distinct responsibility for intervention development and process evaluation as required according to the MRC framework, as well as the necessary expertise, is fulfilled in this study.

### Design and conduct

Key recommendations regarding this phase are as follows: describing the intervention and clarifying causal assumptions, defining the most important research questions, and selecting a combination of methods. In the STADPLAN study, we use a logic model to show the main elements of the intervention and the procedural outcomes we focus on. To define the relevant outcomes, we considered previous research in this domain. Furthermore, we approached important stakeholders in advance, both on local and national levels, for example, the association of legal guardians and the federal nursing council. The advisory board incorporates members representing research in ACP, geriatrics, and primary care as well as a patients’ organization. Thus, we were able to collect expertise and receive support on relevant contextual factors and current considerations in the research field.

The instruments are based on theoretical foundation and the recommendations of stakeholders and target each step of the MRC evaluation framework (see Fig. 3). We will use a mixed methods approach of qualitative and quantitative methods including all defined participants of the intervention and their context. We will collect part of the data in the whole sample (such as assessing the ACP conversations protocol or interviews with the heads of HCS); part will be collected in a subsample. The approach has been successfully applied in previous process evaluation designs [31–33].

### Analysis

Key recommendations of this phase are considering mixed methods data, analyzing these in an iterative process, building qualitative and quantitative analyses on each other, and publishing process data early in advance to main outcome measures. In our pilot study, we already developed the analysis scheme for the qualitative data, building a deductive analysis process upon the concepts incorporated in our logic model.

This allows us to analyze the process data alongside the running trial. We decided to set data collection points for process evaluation in BEVAs, patients, and caregivers around t1 (6 months after baseline), after implementation of the intervention. This will facilitate the timely completion of the process evaluation. At the end of the study, we will conduct interviews with the heads of the HCS for a full account of their experiences.

### Reporting

We will disseminate results via conferences, scientific forums, and publications as well as via reports and workshops targeted towards stakeholders. In reporting this protocol and the results of the process evaluation, we followed the MRC framework and previous study protocols and process evaluations with comparable approaches [34, 35]. In addition, the complex intervention is reported based on the Criteria for Reporting the Development and Evaluation of Complex Interventions (CReDECI 2) [36] (Additional file 1).

After completion of the study, we will invite all stakeholders for a presentation of first results by the universities on a local level, promoting discussion and networking. Thus, we aim to enhance impact of research on the local level of nursing practice.

## Supplementary information

**Additional file 1.** CReDICI 2 checklist STADPLAN project.

## Data Availability

The datasets generated and analyzed during the current study are not publicly available due to conditions of informed consent with participants. Please contact corresponding author in case of reasonable requests.

## Abbreviations

ACP: Advance care planning
BEVA: Facilitator for ACP in the home care setting (German acronym: Begleiterin für vorausschauende Versorgungsplanung in der Ambulanten Pflege)
CG: Control group
CReDECI: Criteria for the Reporting of the Development and Evaluation of Complex Interventions
GP: General practitioner
HCS: Home care service
IG: Intervention group
MRC: Medical Research Council
PAM: Patient Activation Measure
STADPLAN: Study on advance care planning in care-dependent community dwelling older persons
