# Supplementary material for: Process evaluation of a complex intervention to promote advance care planning in community-dwelling older persons (the STADPLAN study)—study protocol
Source: Trials. 2020 Jul 16;21:653. doi: 10.1186/s13063-020-04529-2 (PMC7366302; doi:10.1186/s13063-020-04529-2)
Supplement: Supplementary file 1 — Additional file 1. CReDICI 2 checklist STADPLAN project. [file 13063_2020_4529_MOESM1_ESM.pdf]

Additional file 1: CReDICI 2 checklist STADPLAN project			
Item			Reported on page or in publication
First stage: Development			
1	Description of the intervention’s underlying theoretical basis	Advance Care Planning (ACP) (e.g. Rietjens et al. Definition and recommendations for advance care planning 18(9):e543-e551.)<br>Respecting Choices (e.g. Briggs L, Hammes B. Respecting Choices. Building a Systems Approach to Advance Care Planning 2011)	Study protocol main study (submitted for publication)
2	Description of all intervention components including the reasons for their selection as well as their aims / essential functions	Intervention components:<br>• ACP conversation (core component of ACP: encourage patients to deal with the topic of ACP in a structured way, support communication on ACP in the family and nominating a surrogate decision maker)<br>• Information brochure and workbook (additional information and possibility to re-read for participants and representatives)<br>• Two-day training for nurse facilitators (preparation and education for conducting the intervention)<br>• Written information for the participant´s GP (to sensitize and prepare GPs on questions regarding medical treatment options)	Study protocol main study (submitted for publication)
3	Illustration of any intended interactions between different components	See logic model	Study protocol for the process evaluation (current paper, Fig. 1 and Lines 123 – 185)
4	Description and consideration of the context’s characteristics in intervention modelling	Context characteristics<br>**Participants:** care-dependent community dwellers.<br>Aim: facilitate access to ACP, consider potential frailty and minor cognitive impairment, create comfortable setting for ACP and data collection<br>→intervention takes place at participants’ home, as well as data collection<br>→access to participants via HCS<br>→inclusion of persons of trust / family caregivers<br>→information material suitably adjusted to participants needs (concise, large font, lay language)<br>**HCS (home care services):** scarce funding and staff resources.<br>Aim: facilitate participation	Study protocol main study<br>(submitted for publication)

		→reimbursement for participating HCS, according to workload in CG / IG<br>→educational program of two days, to keep time expenditure feasible<br>**Participating nurses / ACP facilitators:** experienced nurses with 3-year exam, communication skills, established trustful relationship with patients. Aim: develop competencies to conduct intervention by efficient training.<br>→minimum standard of competencies predefined for participating nurses<br>→intensive course, building on existing competencies, but of feasible length (amount of time, content)<br>→topic guides for ACP-conversations, ensuring comparability and fidelity<br>→second day workshop allowing for individual adjustment according to nurses' experiences	
Second stage: Feasibility and piloting			
5	Description of the pilot test and its impact on the definite intervention	**Pilot trial:**<br>A pilot trial was conducted at two study sites, with a total of 4 participating health care services as intervention cluster.<br>Included participants: n=21 (Halle), n=10 (Oldenburg)<br>Participating nurses (BEVAs): n=3 (Halle), n=1 (Oldenburg)<br>ACP-conversations that took place: n=16 (Halle), n=9 (Oldenburg)<br>Tested instruments: CRF t0 and t1, hypothetical scenarios (n=16), process evaluation instruments (interviews, observations, questionnaires: n=16 (Halle) + n=32 (Oldenburg))<br>Duration: 3 months<br><br>**Adjustments:**<br>• Recruitment of participants: more time for HCS, documentation of recruitment efforts, adjustment of inclusion criteria (age 60 instead of 65 years and older); provision of recruitment guidance for HCS (why should clients participate?)<br>• Data collection: data collection points 3 instead of 5, allowing a longer period of data collection; reduction of variables; show cards with responses in questionnaires to support needs of older persons; data collection in HCS shifted towards data collection in participants (data more complete); decision for face-to-face data collection on	Not published

		participants level<br>• Minor revisions on process evaluation instruments<br>• Minor revision: reduction of qualitative data collection in participants (no audiotaping, only protocol of open-ended questions), reduction of data collection in staff (only participating nurses / BEVAs)<br>• Minor revisions on the conversations' topic guide, minor revisions on educational program, (as a result of nurses' feedback in focus groups)	
Third stage: Evaluation			
6	Description of the control condition (comparator) and reasons for the selection	HCS in the control group receive a short information brochure for participating clients in order to warrant optimised usual care<br>Content of the brochure:<br>• Introduction to ACP, surrogate decision making and advance directive documents (condensed)<br>• Glossary of medical and legal terms, contact information on local consultancies	Study protocol main study<br>(submitted for publication)
7	Description of the strategy for delivering the intervention within the study context	The ACP-conversations are delivered by trained nurses of the participating HCS who are known and trusted by participants.<br>Measures to ensure intervention delivery:<br>1. **Educational program** (led by project staff, identical course material, training and course leaders at the three study sites), divided into 7 modules:<br>M1: Introduction of the STADPLAN study<br>M2: Introduction of the topic ACP<br>M3: Practical exercise of the counselling conversations, extensive practise of the guided conversations with partners using different health situations/cases<br>M4: Facilitator's tasks and schedule in the course of the study<br>M5: Reflexion on conversation experiences<br>M6: Special practical training of difficult conversational situations, refresher of knowledge on ACP<br>M7: Feedback and closing of the training<br><br>2. **ACP counselling (divided into 2 parts), topic guides contain:**	Study protocol main study<br>(submitted for publication),<br>Table 1

		• **Part 1**: Information on the project, ACP, aim of the conversations, information on the tasks and features of the surrogate/ representative, information on the written living will, introduction of the written information brochure, preparation of the next conversation: topic and goal, presence of a representative,<br>• **Part 2**: Repeating information on project, ACP and aim of the conversation, introduction of the following topics: attitudes, preferences and values of the participant, reflection on the use of the additional written information and integration of notes, clarification: further conversations requested?<br>**3. Information brochure with workbook, containing:**<br>• Introduction to ACP, surrogate decision making and advance directive documents<br>• Presentation of critical health scenarios along with incapacity<br>• Glossary of medical and legal terms, contact information on local consultancies	
8	Description of all materials or tools used to deliver the intervention	Course material of the educational program:<br>• Printed version of the PowerPoint presentation used in the program<br>• Printed versions of hand-outs, examples for advance directives, blank forms for surrogate designation, brochures on surrogate decision-making and advance directives<br>Conversations topic guides<br>• Printed topic guides for each participating patient of the BEVAs HCS<br>Information brochure with workbook<br>• Copies of the brochure for each participating patient plus extra copies for the use of the BEVA (preparing for conversations)	Study protocol main study (submitted for publication)
9	Description of fidelity of the delivery process compared to the study protocol	Assessment of:<br>• Number and date of ACP conversations<br>• Extend to which topic guides are used (documentation in commentary fields)<br>• Experiences of BEVAs collected in focus group	Study protocol for the process evaluation (current paper): Methods: Fig 3, Process evaluation framework and methods, Line 246 -

			251
10	Description of a process evaluation and its underlying theoretical basis	The process evaluation is planned and conducted according to the MRC framework for the development and evaluation of complex interventions.	Study protocol for the process evaluation (current paper): Methods: Line 111 - 305
11	Description of internal facilitators and barriers potentially influencing the delivery of the intervention as revealed by the process evaluation	We will describe facilitators and barriers based on the constructs inherent to the logic model and on the perspectives of the participants.	Study protocol for the process evaluation (current paper): Methods: Line 278 - 288
12	Description of external conditions or factors occurring during the study which might have influenced the delivery of the intervention or mode of action (how it works)	We will assess external conditions via the participating heads of HCS, BEVAs, patients and caregivers.<br>In the Logic Model, external conditions are summarized as context related to participants and processes.	Study protocol for the process evaluation (current paper, Fig. 1 and Lines 123 - 185
13	Description of costs or required resources for the delivery of the intervention	We will conduct a health economic evaluation, which estimates implementation and intervention costs during the study period. Further, cost implications of the ACP intervention will be explored.<br>The estimation of (i) implementation und intervention costs will be performed from the perspective of the home care services (on organizational level) as well as the German social insurance system (on patient level).<br>Cost implications of the intervention (ii) during the 12-month follow-up, in particular inpatient care costs (hospital as well as short-term nursing home), expenses for rehabilitation services and medical devices, will be determined from the perspective of the German social insurance system (on patient level).<br>Cost implications of the intervention, which (iii) could be expected in following years and therewith after the end of our study will be explored by using hypothetic scenarios (on patient level).	Study protocol main study<br>(submitted for publication)
